# Case Report: Thrombus aspiration and *in situ* thrombolysis via a Guidezilla guide extension catheter in a patient with high-risk pulmonary embolism

**DOI:** 10.3389/fcvm.2024.1340962

**Published:** 2024-03-28

**Authors:** Jing-Wen Ding, Yu-Ang Jiang, Peng Li, Hong-Xiang Xie, Huai-Sheng Ding

**Affiliations:** Department of Cardiovascular Medicine, The Meishan People’s Hospital, Meishan, Sichuan, China

**Keywords:** case report, pulmonary embolism, catheter-directed thrombolysis, thrombus aspiration, Guidezilla catheter

## Abstract

Standard catheter-directed thrombolysis (CDT) and thrombus aspiration are considered potentially promising approaches for reopening the embolism-related pulmonary artery in patients with pulmonary embolism (PE) with high thrombotic burden and deteriorating hemodynamics, especially in those for whom systemic thrombolysis is contraindicated or has failed. However, the constrained accessibility of dedicated catheters has impeded the potential benefits of standard CDT in developing countries. The Guidezilla guide extension catheter (GEC) with a larger diameter and extended length is widely used in challenging coronary anatomy. Nevertheless, there have been few reports on the application of the Guidezilla GEC as a novel option for patients with massive PE when dedicated catheters and surgical thrombectomy are not available. In this case report, we demonstrated that thrombus aspiration and *in situ* thrombolysis through the Guidezilla GEC are applicable to patients with PE in whom systemic thrombolysis is contraindicated, resulting in successful reperfusion and positive clinical outcomes.

## Introduction

Pulmonary embolism (PE) is caused by intraluminal thrombosis resulting in partial or complete occlusion of the pulmonary artery (PA). The European Society of Cardiology (ESC)/European Respiratory Society (ERS) guidelines recommend that the application of catheter-based techniques should be considered for patients with high-risk PE with contraindications to systemic thrombolysis (1). The current research expands the evidence supporting the use of catheter-directed thrombolysis (CDT) as a promising therapy for acute high-risk PE, demonstrating significant improvement in right heart parameters and PA pressure compared to systemic thrombolysis and anticoagulation (2). The present analysis supports the notion that individuals exhibiting a more adverse baseline right ventricle (RV) to left ventricle (LV) ratio, elevated PA systolic pressure, and increased pulmonary artery obstruction manifest the most pronounced enhancements in these specific parameters after undergoing CDT (3). A meta-analysis of small retrospective studies has shown that CDT is associated with lower short-term mortality and exhibits a tendency toward a reduced 1-year mortality benefit compared to anticoagulation (4). Studies focusing on the enhancement of clinical endpoints through CDT are still strongly needed to advance cardiopulmonary health after PE (3).

The Guidezilla (Boston Scientific, Marlborough, MA, USA) guide extension catheter (GEC) is widely applied in complex percutaneous coronary intervention (PCI) for facilitating deep culprit vessel intubation by enhancing the additional backup support of the guiding catheter (5). As a mother-in-child catheter, the Guidezilla GEC has a larger diameter to enable various interventional devices for performing complicated procedures, which may be applicable for massive PE as an alternative in the absence of dedicated techniques and surgical thrombectomy.

Herein, we present the detailed operation of thrombus aspiration and *in situ* thrombolysis through the Guidezilla GEC for a patient with high-risk PE with absolute contraindication of systemic thrombolysis.

## Case report

**Figure 1:**
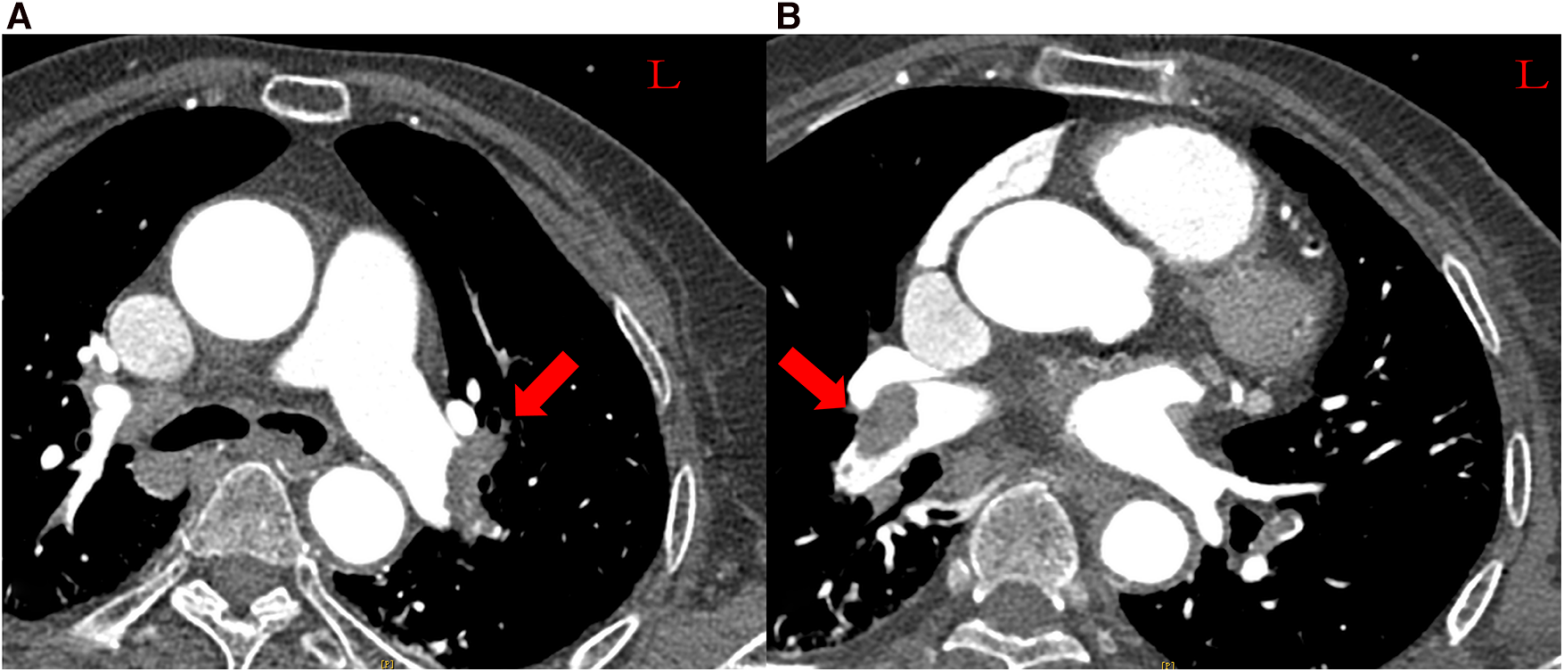
Massive pulmonary embolism. (**A**) Left pulmonary arteries. (**B**) Right pulmonary arteries.

A 70-year-old Chinese female patient with left hydronephrosis was admitted for a planned laparoscopic disconnection of ureteropelvic junction obstruction. For 2 days postoperatively, she developed severe dyspnea and presented with circulatory shock. She had hypotension (79/55 mmHg) on norepinephrine (0.4 µg/kg/min), sinus tachycardia (122 beats/min), tachypnea with 44 breaths/min, and her initial oxygen saturation was 93% on 3 L oxygen and worsened to 81% on 5 L. The elevated D-dimer levels (13.28 µg/mL) suggested a high suspicion of pulmonary embolism. Computed tomography pulmonary angiography (CTPA) confirmed the diagnosis of pulmonary embolism, revealing large emboli in the bilateral pulmonary arteries (Figure 1). As systemic thrombolysis was contraindicated and surgical pulmonary thrombectomy was unavailable in our hospital, the patient was transferred to the Department of Cardiovascular Medicine and taken to the operating room for percutaneous catheter-directed therapy.

**Figure 2:**
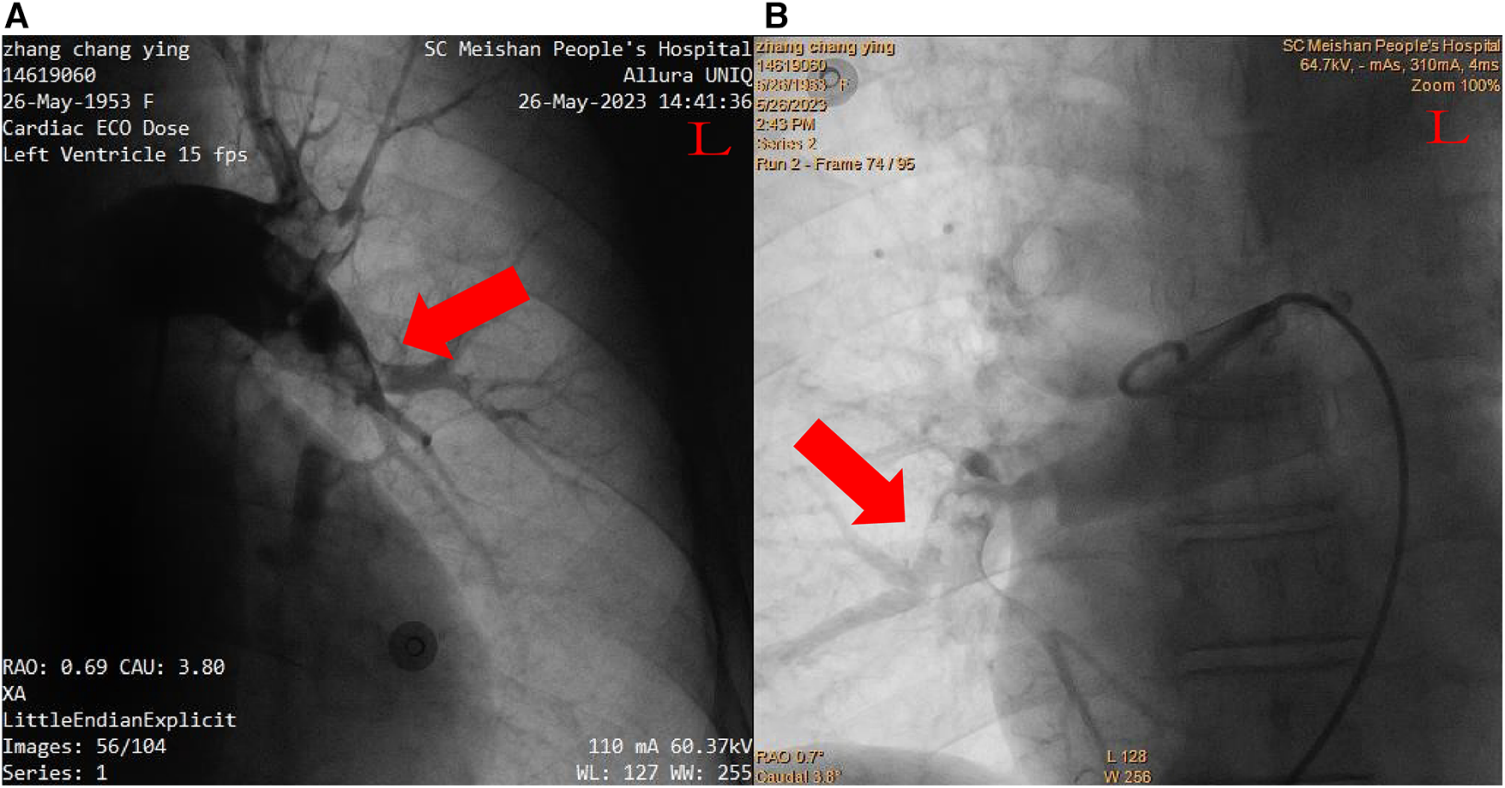
Pulmonary angiogram. (**A**) Left pulmonary arteries. (**B**) Right pulmonary arteries.

**Figure 3:**
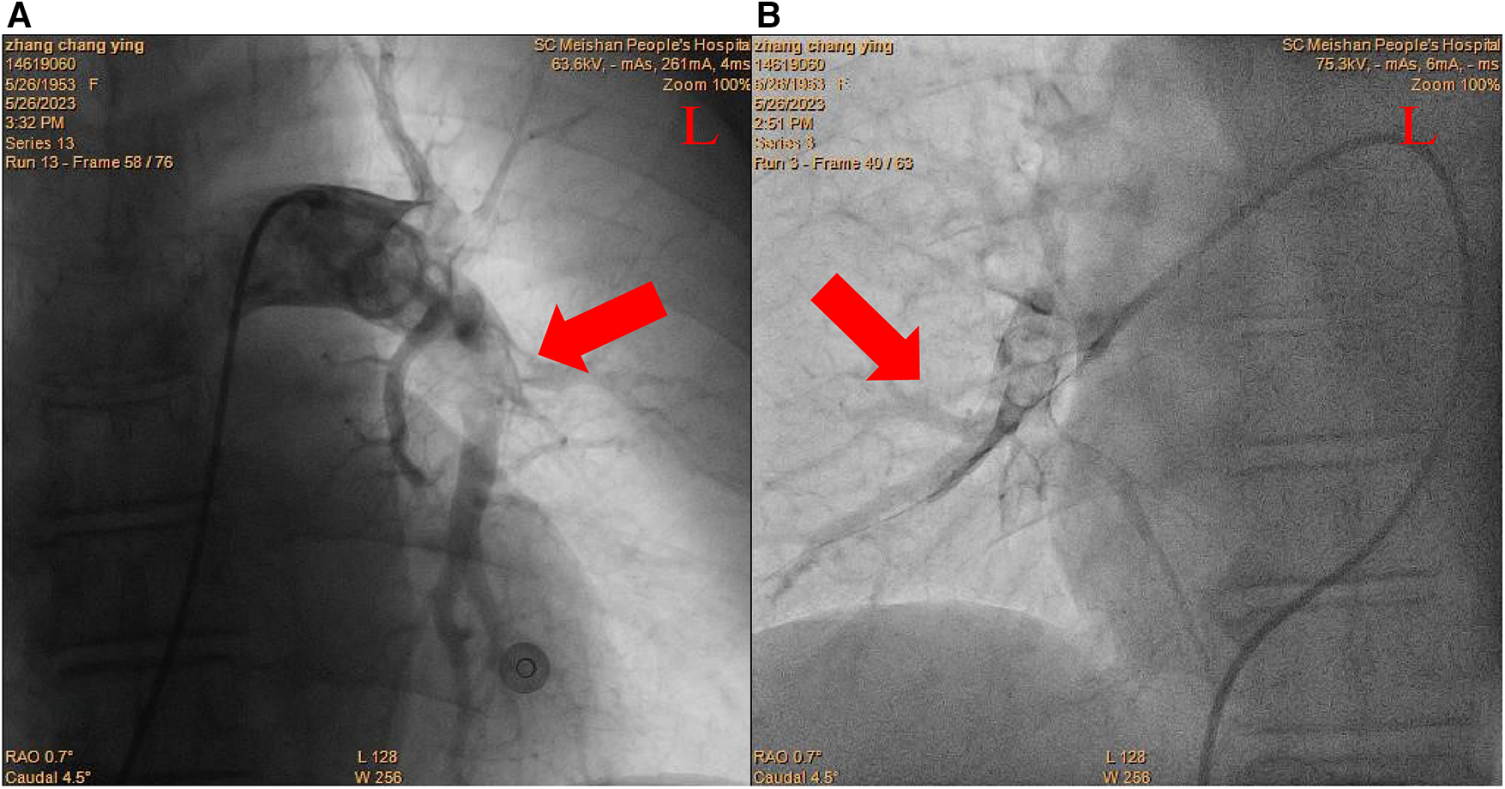
Postcatheter-related treatment. (**A**) Left pulmonary arteries. (**B**) Right pulmonary arteries.

After inserting a 6-French (Fr) flex introducer sheath into the right femoral route, a 6-Fr pigtail catheter was advanced over a 0.035-inch guidewire to reach the pulmonary trunk. The catheter-directed pulmonary angiogram confirmed simultaneous filling defects of the right and left pulmonary arteries, especially of the left PA with large saddle emboli (Figure 2). Since regular percutaneous catheter-directed systems of PE were unavailable in our hospital, the Export Aspiration Catheter (EAC; Medtronic Corporation, Sunnyvale, CA, USA) with 1.37 mm (0.054-inch) aspiration lumen, which is widely used in PCIs for establishing antegrade flow before culprit vessel stenting, was used to remove pulmonary large clots through aspiration mechanisms. After dilatation of the Sprinter balloon (2.5 × 20 mm; Medtronic, Inc., Minneapolis, MN, USA) in the distal part of the right middle-pulmonary artery, the EAC was inserted for thrombus aspiration. With the limited diameter of the EAC, thrombus aspiration seemed to be ineffective, and only a small amount of thrombus was removed. In total, 1 million IU of urokinase was delivered through the EAC, a significantly lower dose than the standard treatment indicated for systemic thrombolysis, with a loading dose of 4,400 IU/kg, followed by 4,400 IU/kg/h over 12–24 h (1). Thus, the Guidezilla GEC with a larger inner diameter (0.067 inches, 1.71 mm), which allows more room for aspiration, was considered to be the optimal alternative to remove the thrombosis clot. Rather unexpectedly, the Guidezilla GEC captured several fresh thrombi in the bilateral PA and successfully aspirated and brought them out from the catheter. Subsequently, 1 million IU of urokinase were bolus injected as *in situ* thrombolytics in the left main pulmonary artery through the Guidezilla GEC. The pigtail catheter-directed pulmonary angiogram demonstrated a significant reduction in the size of the thrombus and the reperfusion in the bilateral PA was substantially improved (Figure 3).

**Figure 4:**
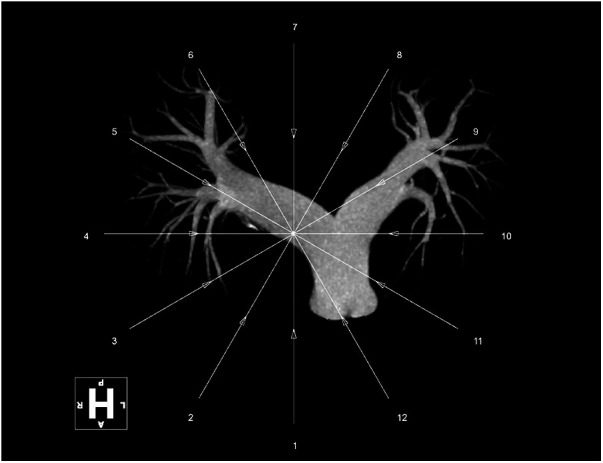
3D reconstruction of CTPA in 2 months later.

Over the next few hours after the procedure, the patient's oxygenation gradually increased to 99% on 2 L oxygen, hemodynamic status was restored to 122/66 mmHg on significantly reduced norepinephrine and a sinus rhythm (73 beats/min) was observed. The severe dyspnea was conspicuously alleviated, and tachypnea was resolved to normal status (19 breaths/min). Five days after the thrombus aspiration and *in situ* thrombolysis through the Guidezilla GEC, the patient's clinical condition was further improved and she was finally discharged to continue her rehabilitation. A new CTPA, scheduled for 2 months later, demonstrated a significant reduction in the size of the thrombus, which was barely visible (Figure 4).

## Discussion

Although rescue thrombolytic therapy is generally recommended for patients with hemodynamic deterioration on anticoagulation treatment, the ESC/ERS guidelines have shown that systemic fibrinolysis can predispose patients to critical hemorrhage if they have undergone surgery in the previous 3 weeks (1). In this case, the elderly patient with massive PE presented an absolute contraindication to fibrinolysis due to the high risk of severe hemorrhage, indicating that the standard CDT appeared to be the preferred alternative reperfusion options.

Notwithstanding the early studies being limited, the severe complications of conventional therapy encouraged the research into novel alternative treatments for reducing the thrombolytic dose. The standard CDT involves advancing the dedicated catheters into the culprit pulmonary artery with thrombus distribution for restoration of pulmonary blood flow by delivering thrombolytics (6). Multiple studies have demonstrated the advantage of immediate thrombus removal through percutaneous catheter-directed thrombectomy and aspiration by providing strong evidence of excellent safety, which may be credited to locally administered low-dose thrombolytics (7, 8). The FLASH trial evaluated the effectiveness of percutaneous catheter-directed thrombectomy and aspiration for the treatment of intermediate to high-risk PE and showed it has a tendency toward superiority in immediate hemodynamic improvements and cardiac function recovery (9).

Different types of dedicated catheters are adopted for mechanical fragmentation, thrombus aspiration or, more commonly, a pharmacomechanical approach combining mechanical or ultrasound fragmentation of the thrombus with *in situ* reduced-dose thrombolysis (10). The UniFuse (AngioDynamics, Latham, NY, USA) and Cragg-McNamara (ev3 Endovascular, Plymouth, MN, USA) 4–5-Fr infusion catheters, with an infusion length of 10–20 cm, are recommended as currently favorable treatment options (1). The overall procedural success rates of thrombus aspiration and *in situ* thrombolysis via different catheter devices are in the range of 70.8%–83.1% (11). However, the actual value of dedicated techniques should be interpreted with caution due to the limited availability of specialized catheters in clinical practice, especially in developing countries. The utilization of dedicated catheters was confined to leading tertiary medical centers in China, potentially limiting the clinical benefits of CDT. The Guidezilla GEC is widely recognized as a frequently used support catheter in chest pain centers in China. It has been designed with a larger diameter for challenging coronary vasculature anatomy to allow the use of various interventional equipment by providing extra deep seating support during complex PCI procedures. Simultaneously, due to its flexibility and widespread utilization in China, this catheter's effectiveness as auxiliary equipment can be extended to thrombus aspiration and the delivery of thrombolytics for massive PE.

To the best of our knowledge, few cases have been reported where local thrombolytics and aspiration were successfully delivered through a Guidezilla GEC. In the present case, a total of 2 million IU of urokinase, a significantly reduced dose than the routine basis indicated for systemic thrombolysis, was delivered in bilateral pulmonary arteries equally after aspiration through the Guidezilla GEC (1). Compared with routine systemic thrombolysis, the delivery of a reduced-dose thrombolytic after thrombus aspiration by the Guidezilla GEC increases its local concentration in the culprit vessels, potentially achieving the most optimal balance between safety and efficacy. The success of the practice of the Guidezilla GEC appeared to be consistent with the dedicated equipment of standard catheter-directed treatment in terms of net clinical benefit. The Guidezilla GEC is one of the most commonly used GECs, which makes it a promising and potentially optimal substitute device from an emergency perspective for massive PE.

## Conclusion

The percutaneous catheter-directed treatment should be an imperative alternative to rescue thrombolytic therapy for patients with massive PE with deteriorating hemodynamics, in whom systemic thrombolysis has failed or is strictly contraindicated. However, the scarcity of dedicated catheters has barricaded the clinical practice of CDT, especially in developing countries. In this letter, the successful practice of the GuideZallia GEC in this particular patient suggests it could enhance the clinical benefits of treatment of PE, and the advantage of efficiency and safety may have the potential to extend to all patients with indications, serving as an easily accessible alternative. Thus, further cohort trials are required to determine whether it could become a new preferred equipment for wide application among patients with high-risk PE.

## Data Availability

The original contributions presented in the study are included in the article/Supplementary Material, further inquiries can be directed to the corresponding author.
